# Effect of multimodal diagnostic approach using deep learning-based automated detection algorithm for active pulmonary tuberculosis

**DOI:** 10.1038/s41598-023-47146-0

**Published:** 2023-11-13

**Authors:** So Yeon Choi, Arom Choi, Song-Ee Baek, Jin Young Ahn, Yun Ho Roh, Ji Hoon Kim

**Affiliations:** 1 Department of Emergency Medicine, Yonsei University College of Medicine, 50-1 Yonsei-Ro, Seodaemun-Gu, Seoul, Republic of Korea; 2 Institute for Innovation in Digital Healthcare, Yonsei University, Seodaemun-Gu, 50 Yonsei-Ro, Seoul, Republic of Korea; 3 Department of Radiology, Research Institute of Radiological Science, Center for Clinical Imaging Data Science, Yonsei University College of Medicine, Severance Hospital, Seoul, Republic of Korea; 4 Division of Infectious Disease, Department of Internal Medicine, Severance Hospital, Yonsei University College of Medicine, Seoul, Republic of Korea; 5 Biostatistics Collaboration Unit, Department of Biomedical Systems Informatics, Yonsei University College of Medicine, Seoul, Republic of Korea

**Keywords:** Diagnosis, Health care economics, Infectious diseases, Respiratory tract diseases

## Abstract

In this study, we developed a model to predict culture test results for pulmonary tuberculosis (PTB) with a customized multimodal approach and evaluated its performance in different clinical settings. Moreover, we investigated potential performance improvements by combining this approach with deep learning-based automated detection algorithms (DLADs). This retrospective observational study enrolled patients over 18 years of age who consecutively visited the level 1 emergency department and underwent chest radiograph and sputum testing. The primary endpoint was positive sputum culture for PTB. We compared the performance of the diagnostic models by replacing radiologists’ interpretations of chest radiographs with screening scores calculated through DLAD. The optimal diagnostic model had an area under the receiver operating characteristic curve of 0.924 (95% CI 0.871–0.976) and an area under precision recall curve of 0.403 (95% CI 0.195–0.580) while maintaining a specificity of 81.4% when sensitivity was fixed at 90%. Multicomponent models showed improved performance for detecting PTB when chest radiography interpretation was replaced by DLAD. Multicomponent diagnostic models with DLAD customized for different clinical settings are more practical than traditional methods for detecting patients with PTB. This novel diagnostic approach may help prevent the spread of PTB and optimize healthcare resource utilization in resource-limited clinical settings.

## Introduction

The World Health Organization (WHO) has identified tuberculosis as the most common life-threatening infectious disease and a leading cause of death worldwide^1,2^. Early detection of pulmonary tuberculosis (PTB) is essential for mitigating the spread, morbidity, mortality of the disease, as well as the burden of care for patients, families, and the overall public health system^1,3–5^. In suspected cases of active PTB, isolation and adherence to airborne precaution guidelines are recommended prior to confirmation, given that confirming the results of culture requires several weeks, which is the gold standard for PTB diagnosis^4,6,7^. Treatment and isolation of a patient, which are necessary as soon as the disease is suspected, could be chosen instead. Therefore, effective strategies are needed to facilitate the prompt diagnosis of active TB in medical institutions in areas with a high burden of the disease^7,8^, as failure to rapidly and accurately identify PTB can result in nosocomial infections or wastage of isolation resources.

In clinical settings, if PTB is suspected based on the patient's clinical manifestations and chest radiography, a sputum test such as a smear microscopy or polymerase chain reaction (PCR) is performed. This is followed by a sputum culture to confirm the diagnosis^7,8^. In addition, chest computed tomography (CT) complements differential diagnosis and guidance for clinical decisions during the treatment for PTB^9,10^. More recently, deep learning-based automated detection algorithms (DLAD) have been introduced for PTB prediction^6,7,11^. However, these diagnostic tools have clear limitations when performing diagnosis prior to confirmation of culture results based on a single test^6,7,9^. Given the differences in available diagnostic tools for PTB between regions and institutions and the uncertainty about the time required to obtain results^3,4^, clinicians should consider the results of only the diagnostic tests performed in a given clinical setting when making decisions, such as administering TB drugs and using isolation resources. However, there is no consensus on strategies for effectively combining the results of different tests for the diagnosis of PTB to support clinical decision making. To the best of our knowledge, no previous studies have addressed this gap in knowledge. Therefore, we aimed to develop a model to predict culture test results for PTB in a multimodal approach using available tests in clinical settings with different diagnostic tools that may be available. Additionally, we sought to determine whether combining our diagnostic model with DLAD, a recently developed TB detection tool, would improve diagnostic performance.

## Method

### Study design and setting

This retrospective observational study was conducted using prospectively collected data from the emergency department (ED) registry. We followed the STROBE guidelines and adhered to the tenets of the Declaration of Helsinki. This study was approved by the institutional review boards of Severance Hospital (approval number 4-2022-0481). Due to the retrospective nature of the study, the need of informed consent was waived by the institutional review boards of Severance Hospital.

In South Korea, approximately 20,000 new cases of TB are diagnosed each year (equivalent 35.7 cases per 100,000 population in 2021), of which approximately 2.5% are hospitalized. South Korea has low TB prevalence, resulting in low pretest probability. The present study was performed at a tertiary hospital with Level 1 ED located in Northwestern Seoul (the capital city of South Korea). Approximately 100,000 patients visit this ED per year.

This ED is currently following a standardized diagnostic protocol for patients with suspected PTB. The sputum for three pairs of smear microscopy, PCR (Gene Xpert MTB/RIF), and Mycobacterium tuberculosis (MTB) sputum cultures on solid and liquid media are obtained from patients with suspected PTB based on chest radiographs and clinical presentation during the initial assessment. The results of smear microscopy are obtained within 4 h; however, owing to a predetermined test reception time, the time required for obtaining results in practice is 24 h. PCR requires approximately 6 h to confirm the results, whereas sputum culture takes more than 6 weeks.

Additionally, a chest CT scan is performed if the physician is unsure of the presence of active disease based on the chest radiograph and clinical presentation or if a cause other than PTB needs to be differentiated. All chest radiographs and CT images performed in the ED are interpreted within 12 h by board-certified radiologists with at least three years of experience.

### Study population and data collection

Our study was conducted on patients over 18 years of age who consecutively visited the ED between January 2018 and December 2021. We included all patients with suspected PTB based on chest radiographs and clinical presentation at the time of visit and who underwent sputum testing (smear microscopy, PCR, sputum culture) in accordance with a standardized diagnostic protocol for PTB.

The present study data were extracted through the Clinical Research Analysis Portal (SCRAP), which is operated by the data portal system at the study site. Based on this data platform, we obtained patient information on the sex age, vital signs, medical history, symptoms, and results of blood tests performed at the time of visit. We also collected chest radiographs and CT readings, as well as the results of sputum testing performed to diagnose PTB.

### Deep learning algorithm for detecting tuberculosis screening score

All chest radiographs used in this study were analyzed using deep learning-based automated detection algorithms (DLAD) for chest radiographs, capable of detecting active cases of PTB; these algorithms are not yet commercially available. The tuberculosis screening score analyzed through this technology (Lunit INSIGHT CXR v3.1.5.0) was collected for the study. This new DLAD is an improvement over previously released DLADs, which predicted the presence/absence of TB by assuming the maximum value of the prediction scores for nodules and consolidations. The new model is more sophisticated and less dependent on other lesions, such as nodules or integration. To develop this new DLAD, chest radiographs with a microbiological reference standard (culture and/or GeneXpert test) were used for training. In the training stage, the model was trained to predict active TB using an additional 140,285 (16,846 positive and 123,439 negatives) data points with TB annotations. The new DLAD met the target product profile criteria for a triage test set forth by the WHO, with a threshold of 0.15 achieving 70% specificity and the corresponding sensitivity. In the screening setting, compared to the normal cases without any abnormal findings, the performance test of the new DLAD showed an area under the receiver operating characteristic curve (AUROC) of 0.984, a sensitivity of 93.78%, and a specificity of 95.56%. Furthermore, in the triage setting, where all cases containing normal and abnormal findings were included, the results showed an AUCROC of 0.928, a sensitivity of 93.78%, and a specificity of 70.85%. The probability score for the high-sensitivity cut-off used in this test was 0.15^12^.

### Outcome measures

The primary endpoint of this study was the confirmation of PTB. A positive result is defined as the growth in MTB, which serves as the reference standard for active PTB^4,6,7^. Radiologic examination results are defined as positive if interpreted as suspicious for active TB by a radiologist, whereas the results are considered negative if interpreted as non-tuberculous mycobacteria (NTM) or old TB lesions. The TB screening score quantified in the DLAD is measured as a continuous variable ranging from 0 to 100.

### Model development

The entire dataset was randomly split into training and test sets in a 7:3 ratio. We developed a model to diagnose PTB using a training dataset. First, we analyzed the factors that were significantly associated with a positive culture result of PTB among the variables of past history, clinical symptoms, and blood test results through univariable logistic regression. Subsequently, based on a combination of the 8 factors identified through univariable analysis and 4 diagnostic tests for TB, a total of 10 diagnostic models were developed. The combinations of diagnostic tests were organized sequentially based on increasing input variables considering the time required to confirm the results, and five additional models were developed for the same model when the interpretation of chest radiography was replaced with the DLAD. In addition to the 10 nested models accounting for clinical relevance, we further developed a diagnostic model with multivariate logistic regression using the Akaike information criterion (AIC) stepwise selection method. All developed models were validated using the test dataset.

### Statistical analyses

Categorical variables were reported as counts and percentages, and continuous variables were expressed as the mean and standard deviation. For baseline comparisons, we used the student T-test for continuous variables and Fisher’s exact test or chi-square test for categorical variables.

We evaluated the predictive performance including sensitivity, specificity, accuracy, positive predictive value, negative predictive value, and AUROC for each diagnostic test for PTB. Univariable analyses were performed using logistic regression with variables associated with PTB based on previous studies. We obtained odds ratio with 95% confidence intervals (CIs) and *p*-values. Each variable associated with a *p*-value below 0.1 in the univariable analysis was entered into the multivariable logistic regression models. Thereafter, we calculated the AIC and concordance index of the developed multivariable models. To facilitate the clinical application of these models, we developed a nomogram for the prediction of a positive PTB test, and specificity was calculated with the sensitivity of each model fixed at 90% or higher. In the nested models, the AUROC comparison was performed using the nonparametric bootstrap method when replacing chest radiograph readings with DLAD. The mean and confidence interval of the AUROC difference from 1000 bootstrap samples were presented, and a significant difference was considered if the confidence interval did not include zero. *P* values less than 0.05 were considered to be statistically significant. All analyses were performed using R (package version 4.0.3).

## Results

**Figure 1:**
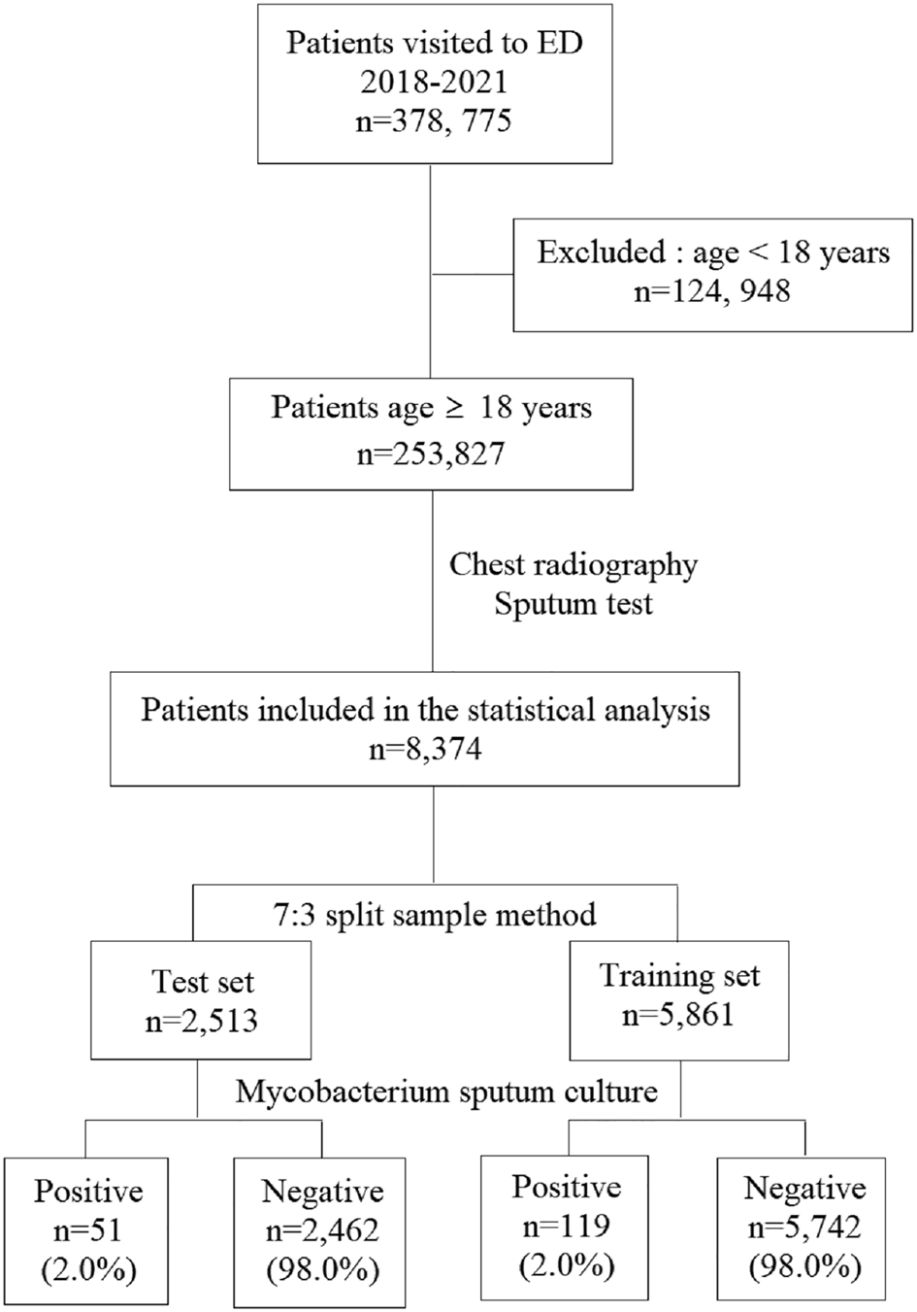
Flowchart of patient enrollment. ED, Emergency Department.

During the study period, a total of 378,775 patients visited the ED, of which 253,827 were aged 18 years or older. Of these, 8,374 patients who underwent sputum testing performed in accordance with the standardized diagnostic protocol for PTB were included in the statistical analyses. In the training set and test set, the number of patients with sputum culture-confirmed PTB was 119 and 51, respectively, accounting for 2% of all patients (Fig. 1). The baseline characteristics and missing rate between the dataset are listed in Table 1. In the study population, 980 and 6222 patients did not undergo chest CT and PCR results, respectively, with missing rates of 23.6% and 74.3%. The body mass index was unknown for 4485 (53.6%) patients.

**Table 1:** Baseline characteristics between training and test set.

Variable	Missing, n (%)	Test set n (%) or mean ± SD (N = 2513)	Training set n (%) or mean ± SD (N = 5861)	*P*-value
Sex	0 (0.0)			0.203
Female		959 (38.2)	2149 (36.7)	
Male		1554 (61.8)	3712 (63.3)	
Age (years)	0 (0.0)	67.5 ± 15.4	67.9 ± 15.6	0.274
Mean arterial blood pressure (mmHg)	0 (0.0)	91.0 ± 20.0	91.2 ± 20.7	0.626
Heart rate (min^-1^)	68 (0.8)	99.9 ± 21.1	100.9 ± 21.7	0.056
Respiratory rate (min^-1^)	70 (0.8)	19.7 ± 4.3	19.9 ± 4.4	0.094
Body temperature (°C)	57 (0.7)	37.3 ± 1.0	37.3 ± 1.0	0.336
Saturation (%)	94 (1.1)	94.8 ± 5.8	94.7 ± 5.6	0.492
Hypertension	0 (0.0)	1166 (46.4)	2773 (47.3)	0.457
Diabetes	0 (0.0)	726 (28.9)	1743 (29.7)	0.450
AIDS	0 (0.0)	3 (0.1)	31 (0.5)	0.012
Old tuberculosis	0 (0.0)	322 (12.8)	788 (13.4)	0.456
Alcohol history	654 (7.8)	1238 (53.5)	2959 (54.7)	0.330
Never smoker	654 (7.8)	1129 (48.8)	2590 (47.9)	0.494
Cough	0 (0.0)	1109 (44.1)	2492 (42.5)	0.180
Sputum	0 (0.0)	1055 (42.0)	2442 (41.7)	0.807
Fever	0 (0.0)	1366 (54.4)	3143 (53.6)	0.554
Dyspnea	0 (0.0)	1603 (63.8)	3796 (64.8)	0.405
Chest pain	0 (0.0)	458 (18.2)	1057 (18.0)	0.860
Hemoptysis	0 (0.0)	337 (13.4)	827 (14.1)	0.416
Anorexia	0 (0.0)	122 (4.9)	265 (4.5)	0.542
General weakness	0 (0.0)	238 (9.5)	552 (9.4)	0.972
Sweating	0 (0.0)	10 (0.4)	22 (0.4)	1.000
Weight loss	0 (0.0)	17 (0.7)	51 (0.9)	0.440
Body mass index (kg/m^2^)	4485 (53.6)	22.2 ± 3.9	22.2 ± 4.1	0.957
Albumin (g/dL)	18 (0.2)	3.3 ± 0.6	3.3 ± 0.6	0.813
High density lipoprotein (mg/dL)	7566 (90.4)	36.4 ± 13.3	35.3 ± 13.8	0.269
Low density lipoprotein (mg/dL)	7729 (92.3)	73.0 ± 35.7	77.7 ± 39.7	0.163
Serum sodium (mmol/L)	11 (0.1)	137.3 ± 4.6	137.4 ± 4.6	0.454
Serum potassium (mmol/L)	10 (0.1)	4.2 ± 0.5	4.2 ± 0.5	0.957
Serum chloride (mmol/L)	12 (0.1)	100.6 ± 5.0	100.8 ± 4.9	0.218
Smear microscopy	0 (0.0)			0.675
Negative		2459 (97.9)	5745 (98.0)	
Positive		54 (2.1)	116 (2.0)	
Polymerase chain reaction^a^	6222 (74.3)			0.506
Negative		609 (99.2)	1529 (99.5)	
Positive		5 (0.8)	9 (0.6)	
Chest radiography	0 (0.0)			0.763
Negative		2500 (99.5)	5840 (99.6)	
Positive		13 (0.5)	21 (0.4)	
Chest computed tomography	980 (23.6)			0.911
Negative		1780 (92.6)	4154 (92.9)	
Positive		142 (7.4)	318 (7.1)	
TB screening score by DLAD	0 (0.0)	19.8 ± 22.5	19.3 ± 22.4	0.327

^a^Polymerase chain reaction (Gene Xpert MTB/RIF).

SD: Standard Deviation, AIDS: Acquired Immune Deficiency Syndrome, TB: tuberculosis, DLAD: Deep Learning-based Automated Detection algorithm.

Our study evaluated the performance of PTB diagnostic tests individually, and the results are presented in Table 2. Smear microscopy and PCR alone were only 41.2% and 22.6% sensitive, respectively, for detecting TB culture. The sensitivity of TB detection based solely on chest radiograph interpretation was 3.4%. Moreover, the cut-off point of the score maximizing the diagnostic performance of DLAD-based TB detection was 20.59, and the sensitivity obtained using this score was 70.6%. The AUROC for detecting TB in chest CT interpretations was 0.759 (95% CI 0.747–0.772), the highest of any single diagnostic modality.

**Table 2:** Diagnostic performance of individual tests for pulmonary tuberculosis detection.

Diagnostic test	Sensitivity	Specificity	Accuracy	PPV	NPV	AUROC
	(95% CI)	(95% CI)	(95% CI)	(95% CI)	(95% CI)	(95% CI)
Chest radiography	0.034	0.997	0.19	0.98	0.977	0.515
	(0.001–0.066)	(0.996–0.998)	(0.023–0.358)	(0.977–0.984)	(0.974–0.981)	(0.503–0.528)
Chest computed tomography	0.58	0.939	0.16	0.991	0.932	0.759
	(0.476–0.683)	(0.932–0.946)	(0.120–0.201)	(0.988–0.994)	(0.925–0.939)	(0.747–0.772)
Smear microscopy	0.412	0.988	0.422	0.988	0.977	0.7
	(0.323–0.500)	(0.986–0.991)	(0.333–0.512)	(0.985–0.991)	(0.973–0.980)	(0.688–0.712)
Polymerase chain reaction^a^	0.226	0.999	0.778	0.984	0.983	0.612
	(0.079–0.373)	(0.997–1.001)	(0.506–1.049)	(0.978–0.991)	(0.977–0.990)	(0.588–0.637)
DLAD	0.706	0.712	0.048	0.992	0.711	0.709
	(0.624–0.788)	(0.700–0.723)	(0.038–0.058)	(0.989–0.994)	(0.700–0.723)	(0.697–0.720)

^a^Polymerase chain reaction (Gene Xpert MTB/RIF).

PPV: Positive Predictive Value, NPV: Negative Predictive Value, AUROC: Area Under the Receiver Operating characteristic Curve, CI: Confidence Interval, DLAD: Deep Learning-based Automated Detection algorithm.

**Figure 2:**
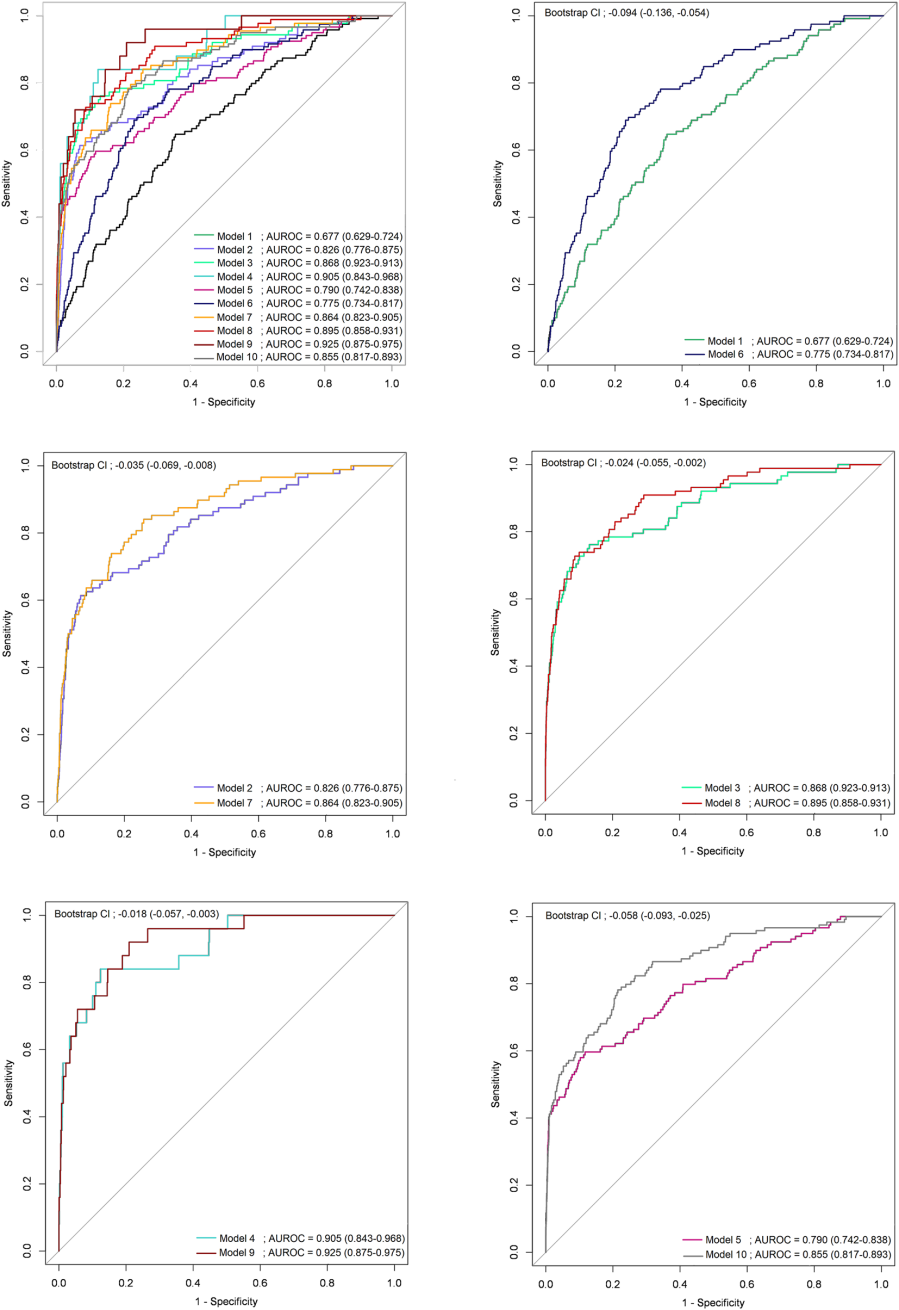
Change in AUROC of nested multicomponent diagnostic models when chest radiograph interpretations are replaced with DLAD by radiologist. AUROC, Area Under the Receiver Operating Characteristic curve; DLAD, Deep Learning-based Automated Detection algorithm; CI, Confidence Interval.

**Figure 3:**
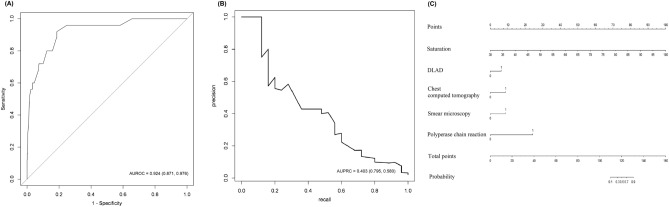
Performance and nomogram of the optimal diagnostic model created using the stepwise selection method. AUROC, Area Under the Receiver Operating Characteristic curve; AUPRC, Area Under Precision Recall Curve; DLAD, Deep Learning-based Automated Detection algorithm.

The eight variables that were significantly associated with PTB in univariable analyses and included in the multicomponent diagnostic model were the respiratory rate, oxygen saturation, dyspnea, anorexia, general weakness, weight loss, albumin, and sodium (Supplement Table S1). The performance of the 10 nested multicomponent diagnostic models, created by combining these 8 factors with the diagnostic tests for TB detection, is shown in Table 3. As additional diagnostic tests were included in the multicomponent diagnostic model, the AUROC and area under precision recall curve (AUPRC) expectably increased, and the clinical factors identified in the univariable analysis lost statistical significance. Chest radiography was not significant as an independent factor in the multicomponent model with other diagnostic tests added; however, the *p*-value for the odds ratio of DLAD to outcome was less than 0.05 in all multicomponent diagnostic models (Supplement Table S2). When the interpretation of chest radiography was replaced by DLAD, except for the models that included all tests, all models showed a statistically significant increase in their AUROC. In other words, if only all tests are available, the use of chest radiography gives equivalent result to DLAD (Fig. 2). Figure 3 plots the performance and nomogram of the optimal diagnostic model created using the stepwise selection method for PTB detection. The optimal diagnostic model had an AUROC of 0.924 (95% CI 0.871–0.976) and an AUPRC of 0.403 (95% CI 0.195–0.580).

**Table 3:** Performance of the 10 nested multicomponent diagnostic models created by combining 8 clinical factors with the diagnostic tests for pulmonary tuberculosis detection.

	AIC	AUROC (95% CI)	AUPRC (95% CI)
Model 1: 8 Clinical factors^a^ + Chest radiography	1122.987	0.677 (0.629–0.724)	0.060 (0.039–0.100)
Model 2: Model 1 + Chest computed tomography	696.491	0.826 (0.776–0.875)	0.172 (0.121–0.257)
Model 3: Model 2 + Smear microscopy	617.011	0.868 (0.823–0.913)	0.379 (0.304–0.481)
Model 4 : Model 3 + Polymerase chain reaction^b^	182.769	0.905 (0.843–0.968)	0.411 (0.177–0.620)
Model 5: Model 1 + Smear microscopy	899.302	0.790 (0.742–0.838)	0.301 (0.223– 0.394)
Model 6: 8 Clinical factors^a^ + DLAD	1055.678	0.775 (0.734–0.817)	0.071 (0.052–0.090)
Model 7: Model 6 + Chest computed tomography	674.281	0.864 (0.823–0.905)	0.210 (0.155–0.284)
Model 8: Model 7 + Smear microscopy	605.789	0.895 (0.858–0.931)	0.385 (0.240–0.476)
Model 9: Model 8 + Polymerase chain reaction^b^	177.128	0.925 (0.875–0.975)	0.412 (0.243–0.609)
Model 10: Model 6 + Smear microscopy	867.581	0.855 (0.817–0.893)	0.311 (0.235–0.414)

^a^8 Clinical factors: respiratory rate, saturation, dyspnea, anorexia, general weakness, weight loss, albumin, serum sodium.

^b^Polymerase chain reaction (Gene Xpert MTB/RIF).

AIC: Akaike Information Criterion, AUROC: Area Under the Receiver Operating characteristic Curve, AUPRC: Area Under Precision Recall Curve, CI: Confidence Interval, DLAD: Deep Learning-based Automated Detection algorithm.

Of the five multicomponent models with conventional interpretations of chest radiography, none had a specificity above 70% when sensitivity was fixed at 90%, whereas two models with DLAD exhibited a specificity above 70%. The optimal diagnostic model created using the stepwise selection method rather than the nested model maintained a specificity of 81.4% when sensitivity was fixed at 90% (Table 4). The calibration plots for multicomponent diagnostic models are shown in Supplement Fig. S3. *P* values for the Hosmer–Lemeshow test in all multicomponent diagnostic models were greater than 0.05, suggesting that diagnostic models were well calibrated.

**Table 4:** Specificity of each multicomponent diagnostic model with 90% sensitivity fixed.

	Sensitivity	Specificity
Model 1 : 8 Clinical factors^a^ + Chest radiography	0.899	0.242
Model 2 : Model 1 + Chest computed tomography	0.898	0.416
Model 3 : Model 2 + Smear microscopy	0.898	0.537
Model 4 : Model 3 + Polymerase chain reaction^b^	0.920	0.552
Model 5 : Model 1 + Smear microscopy	0.899	0.361
Model 6 : 8 Clinical factors^a^ + DLAD	0.899	0.382
Model 7 : Model 6 + Chest computed tomography	0.898	0.547
Model 8 : Model 7 + Smear microscopy	0.898	0.707
Model 9 : Model 8 + Polymerase chain reaction^b^	0.920	0.735
Model 10 : Model 6 + Smear microscopy	0.899	0.514
Optimal diagnostic model	0.920	0.814

^a^8 Clinical factors: respiratory rate, saturation, dyspnea, anorexia, general weakness, weight loss, albumin, serum sodium.

^b^Polymerase chain reaction (Gene Xpert MTB/RIF).

DLAD: Deep Learning-based Automated Detection algorithm.

## Discussion

The present study devised multi-component diagnostic models that are applicable to individualized clinical settings; this strategy will help guide clinical decisions regarding the presence or absence of PTB. Given that all diagnostic test results for PTB were available, more accurate predictions could be obtained; nevertheless, clinical decisions should be optimal even in their absence. Depending on the community and healthcare setting, the distribution of physical and systemic resources for PTB testing varies widely^1,3,4,13^. Consequently, different clinical areas implement different types of diagnostic tests, and the time taken by a physician to assess the results of the same test varies^3,4,13^. In particular, EDs represent clinical settings where patients with acute, uncertain diagnoses may stay for long periods of time, often in close contact owing to crowding. Thus, they are at a higher risk of tuberculosis than patients in outpatient settings^14–16^. Moreover, sputum culture results can take several weeks to confirm, and other diagnostic tests are staggered. In the absence of sufficient reference materials, the decision to isolate and initiate treatment for a patient with suspected PTB has been based so far on clinical experience. The clinical tools developed in our study, which are customized for different clinical settings, can assist physicians in making quantitative and evidence-based decisions.

In the present study, individual diagnostic tests for PTB had poor sensitivity in comparison with specificity. In particular, chest radiographs and smear microscopy, which are conventional tools used for PTB screening, had a sensitivity of less than 50%, which is consistent with the results of previous studies^17,18^. Single prediction using PCR results, which are available in a shorter time frame than smear microscopy^3,19^, also had a low sensitivity for TB detection (22.6%). The low sensitivity of TB detection in healthcare facilities can be related to the spread of nosocomial infections; this implies that TB cannot be ruled out based on a negative test result. Our results suggest that single-test screening approaches are risky for nosocomial transmission, especially in high-density settings such as EDs and multi-bed wards. In this regard, Cattamanchi et al. demonstrated in a prospective cluster trial that a multi-component strategy for the diagnosis of PTB significantly increased diagnosis rates^8,18^. Furthermore, this suggests that a multicomponent diagnostic model for PTB is accurate and beneficial for controlling hospital infections. Increasing the number of diagnostic tests improves accuracy and specificity, while maintaining 90% sensitivity, aligning with WHO guidelines for TB screening^1,2^. Therefore, ensuring rapid turnaround times for multiple diagnostic tests in hospitals is crucial for preventing the spread of nosocomial PTB infection.

Notably, the present study demonstrated that the contribution of the DLAD to the detection of PTB was significantly higher than the interpretation of the chest radiography performed by the radiologist. Chest radiography is valuable for clinically diagnosing PTB and has been a pivotal tool in TB control for over a century, particularly in high-burden clinical setting^17,20,21^. However, the use of chest radiography to detect PTB is limited as this imaging technique lacks accuracy and requires radiological expertise^11,17,21–23^. Chest CT also requires specific expertise, and its limited availability, radiation hazards, and use of contrast media hinder its widespread adoption^17^. Recently, there has been renewed interest in using chest radiography for TB screening, leveraging advances in machine learning approaches to automate chest radiography interpretation^21^. WHO updated their TB screening guidelines to recommend computer-assisted detection software instead of human readers for digital chest radiography analysis for tuberculosis screening and triage of individuals aged 15 years and above^11^. Because DLAD diagnostic performance varied by population in individual previous studies, the high performance of DLAD for single use is not generalizable^20,23,24^. Our study simply confirms the superior sensitivity of DLAD use compared to single use of conventional chest radiography interpretations. Especially, conventional chest radiography interpretations in the multi-component approach were not statistically significant; however, the DLAD remained a significant factor in all models. We also found that replacing conventional strategies with the DLAD significantly improved performance in all multi-component models that could be used when PCR testing was not available. Therefore, the use of the DLAD in combination with other diagnostic tests may be an alternative in clinical settings where advanced diagnostic facilities for the detection of PTB are not available or where the turnaround time for the results is protracted. This finding suggests that our strategy may be particularly helpful in low-income countries where availability for screening for PTB is lacking^5,13,25^.

Globally, the occurrence of PTB is concentrated in underdeveloped countries with limited health care resources, which hinders diagnoses and follow-ups on the disease^13^. Owing to these epidemiological characteristics, the utilization of culture tests as a reference standard in research is rendered a difficult task because of the time required to confirm results^6,21,23,24,26^. Our study was performed at a level 1 ED located in a tertiary hospital with a standardized care protocol for suspected PTB patients, which allowed us to establish a structured cohort from the outset and follow up without data loss until culture results were available. In addition, the study population for tuberculosis-related research is generally imbalanced because it is not highly prevalent. Therefore, previous studies have recommended measuring performance with AUPRC or a framework that specifies target sensitivity and evaluates specificity rather than AUROC^24,27^, and the performance of our diagnostic models was presented using these recommended metrics.

Our study has several limitations. First, our study was conducted in a retrospective design at a single institution, which may limit the generalizability of the findings to other healthcare settings. This is because it contains the potential biases of retrospective studies and our results therefore need to be prospectively validated in study sites with different clinical settings. Second, although study participants were tested in accordance with a standardized protocol, the tested population featured missing cases of PCR testing and chest CT, which introduces bias in the diagnostic performance of the model.

## Conclusions

In conclusion, a multicomponent diagnostic model using various clinical manifestations and ancillary test results is more accurate in detecting active patients with PTB than the diagnostic tools that use a single test. Among these diagnostic techniques, the TB screening score obtained from DLAD as an adjunctive tool for chest radiography can replace traditional interpretations reported by radiologists. Thus, diagnostic models using DLAD can assist in preventing the spread of PTB in resource-limited clinical settings and in optimizing healthcare resource utilization.

### Ethical approval

This study was approved by the institutional review boards of Severance Hospital (approval number 4-2022-0481) and the requirement for informed consent from patients was waived owing to the study’s retrospective design.

### Supplementary Information

Supplementary Figure S3.

Supplementary Table S1.

Supplementary Table S2.

## Data Availability

The datasets analyzed during the current study are available from the corresponding author on reasonable request.
