# Supplementary material for: Effect of multimodal diagnostic approach using deep learning-based automated detection algorithm for active pulmonary tuberculosis
Source: Sci Rep. 2023 Nov 13;13:19794. doi: 10.1038/s41598-023-47146-0 (PMC10643438; doi:10.1038/s41598-023-47146-0)

Supplement Figure S3.
Calibration plot of multicomponent diagnostic models

(A) Model 1

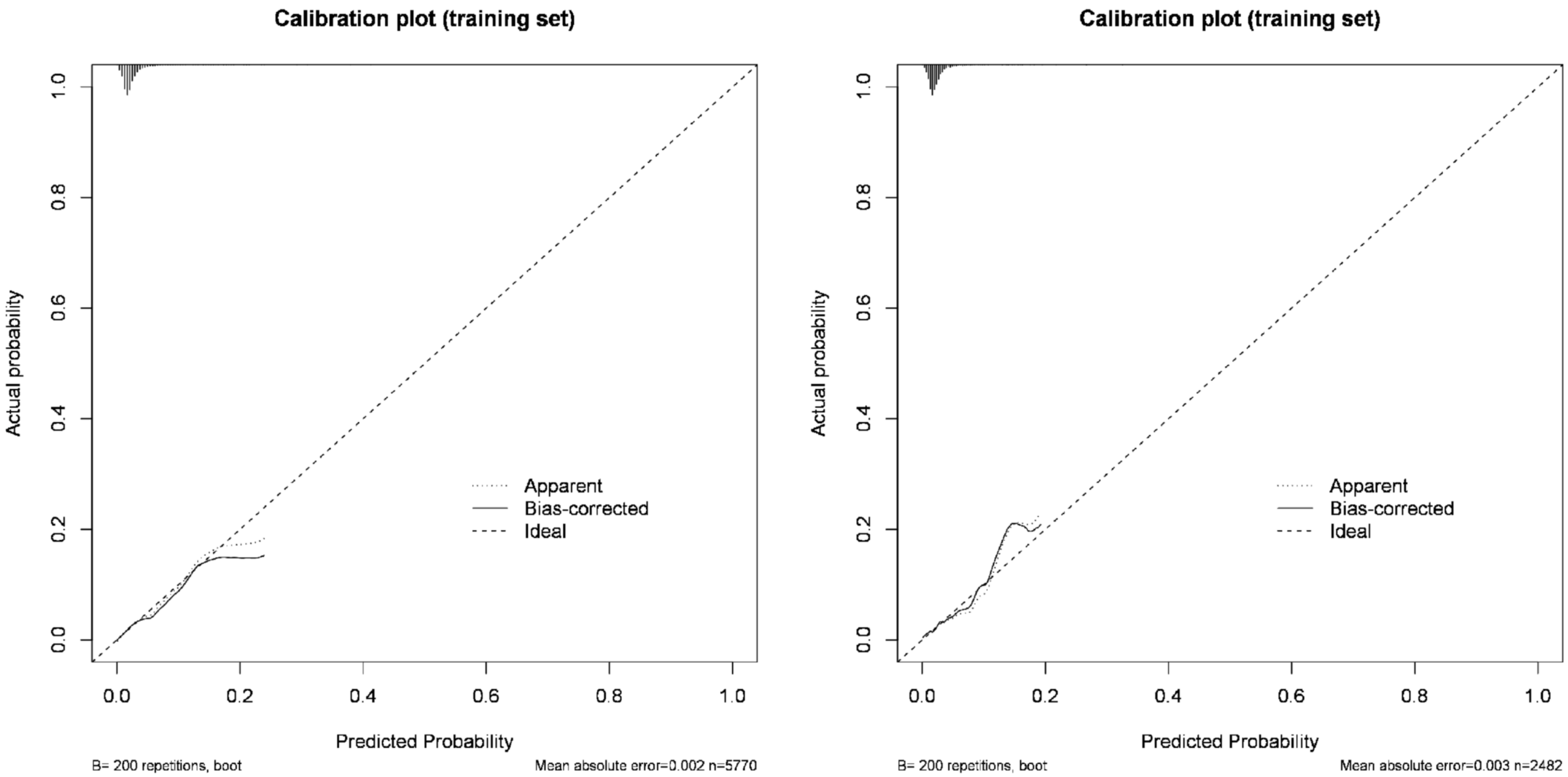
Calibration plot (training set)
Actual probability
Predicted Probability
Apparent
Bias-corrected
Ideal
0.0
0.2
0.4
0.6
0.8
1.0
B= 200 repetitions, boot
Mean absolute error=0.002 n=5770
Calibration plot (training set)
Actual probability
Predicted Probability
Apparent
Bias-corrected
Ideal
B= 200 repetitions, boot
Mean absolute error=0.003 n=2482

(B) Model 2

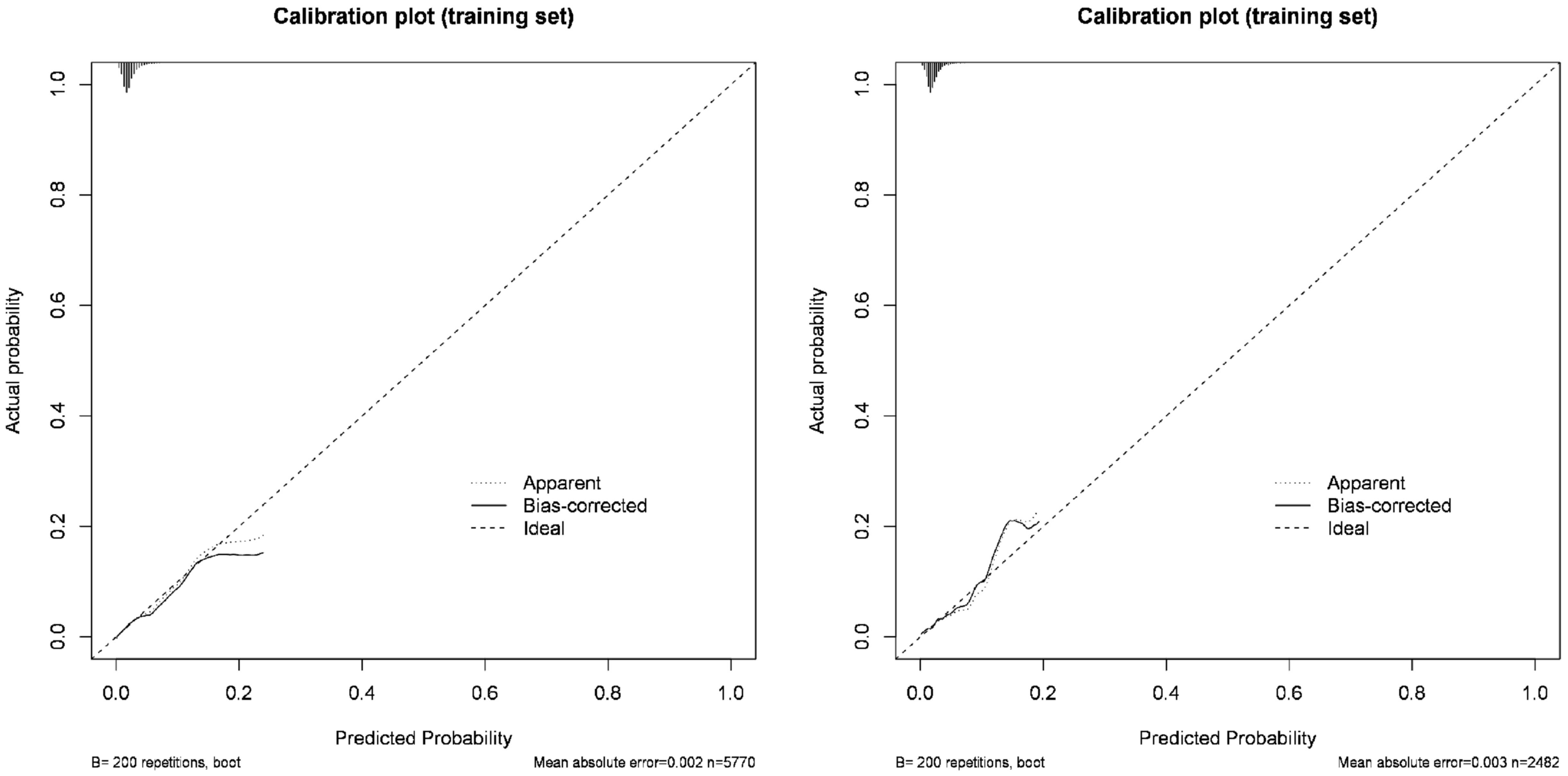
Calibration plot (training set)
Actual probability
Predicted Probability
Apparent
Bias-corrected
Ideal
0.0
0.2
0.4
0.6
0.8
1.0
B= 200 repetitions, boot
Mean absolute error=0.002 n=5770
Calibration plot (training set)
Actual probability
Predicted Probability
Apparent
Bias-corrected
Ideal
B= 200 repetitions, boot
Mean absolute error=0.003 n=2482

(C) Model 3

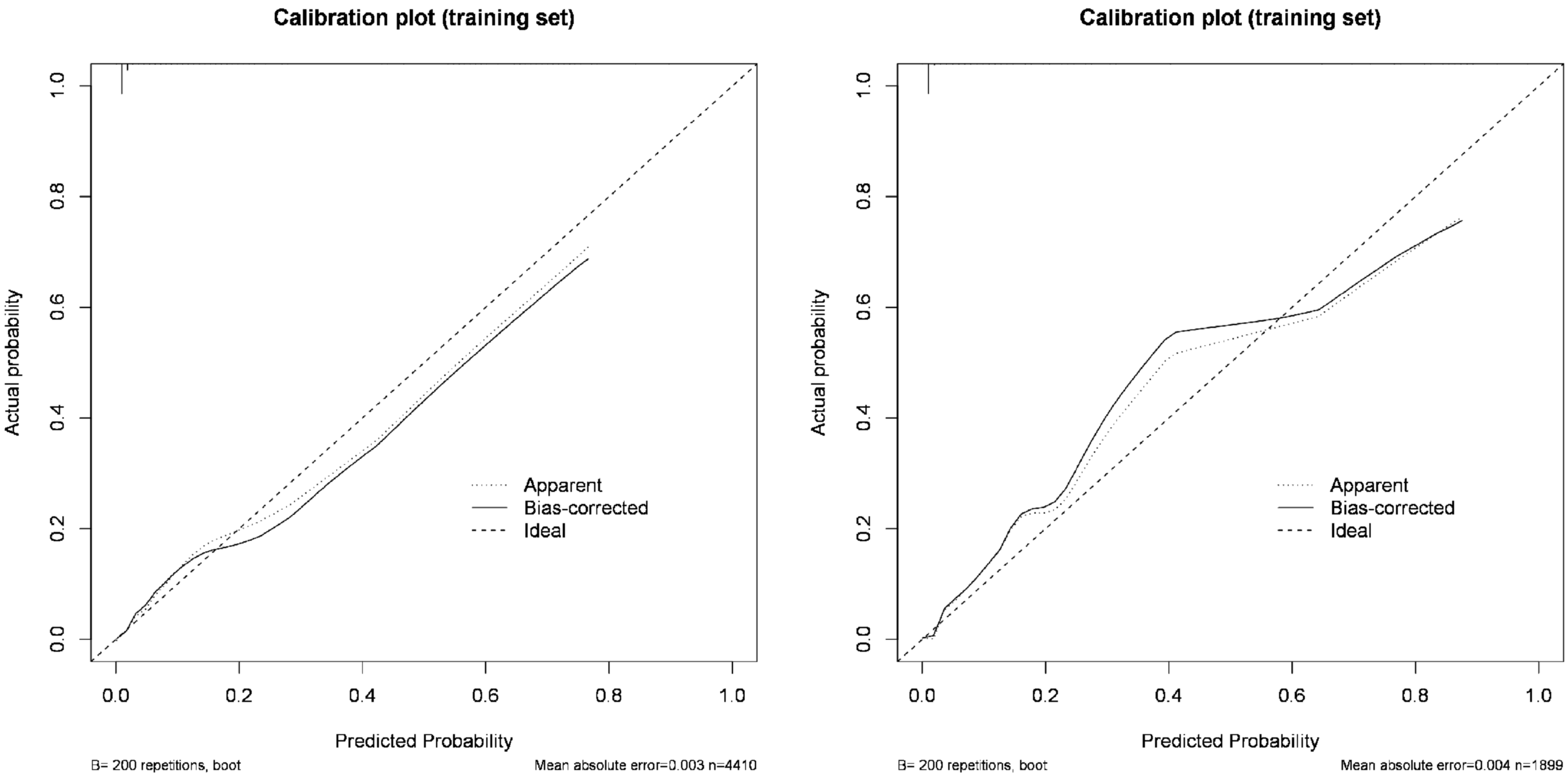
Calibration plot (training set)
Actual probability
0.0
0.2
0.4
0.6
0.8
1.0
Predicted Probability
Apparent
Bias-corrected
Ideal
B= 200 repetitions, boot
Mean absolute error=0.003 n=4410
Calibration plot (training set)
Actual probability
Predicted Probability
Apparent
Bias-corrected
Ideal
B= 200 repetitions, boot
Mean absolute error=0.004 n=1899

(D) Model 4

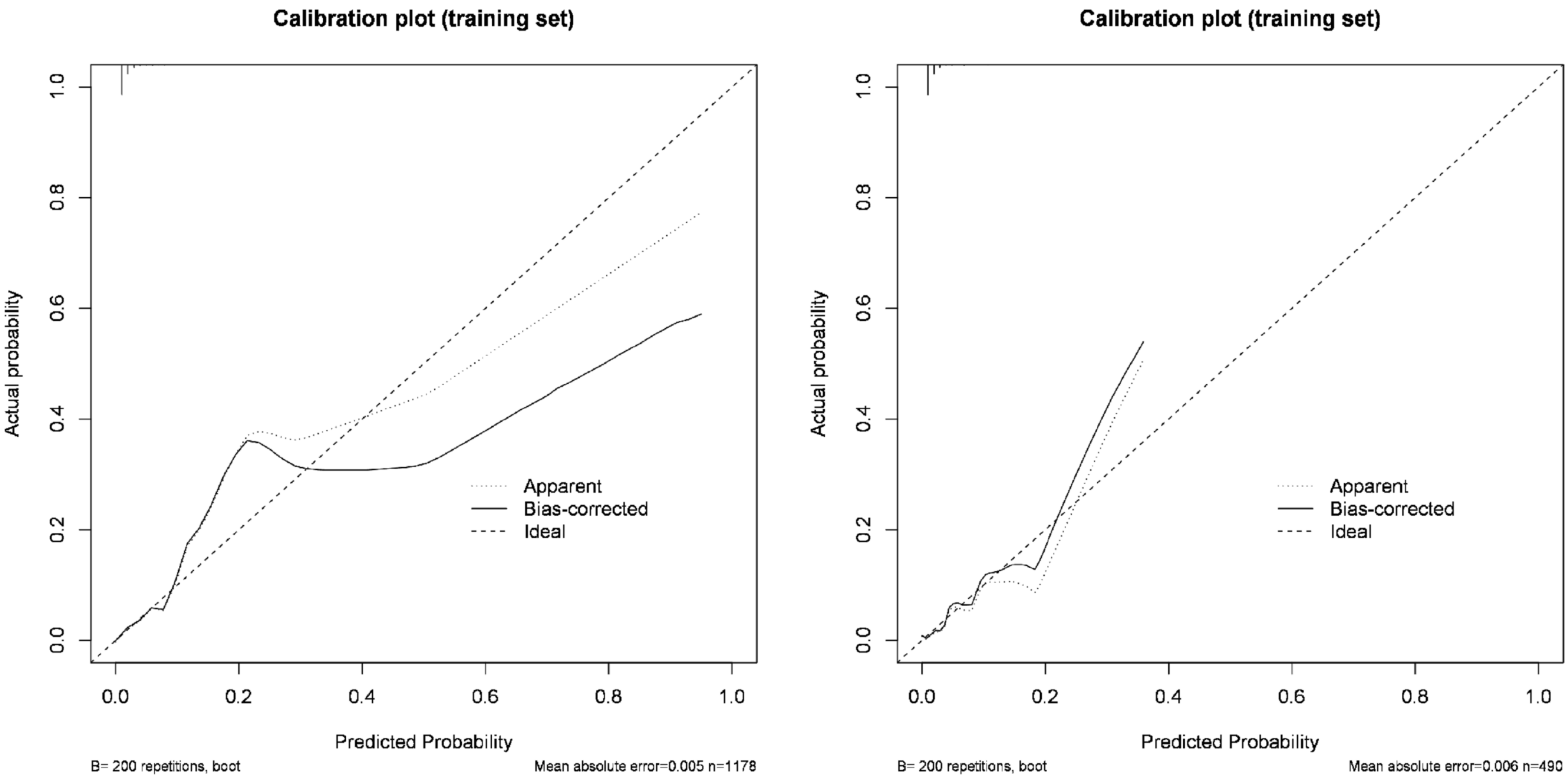

Calibration plot (training set)
Actual probability
Predicted Probability
0.0
0.2
0.4
0.6
0.8
1.0
Apparent
Bias-corrected
Ideal
B= 200 repetitions, boot
Mean absolute error=0.005 n=1178
Calibration plot (training set)
Actual probability
Predicted Probability
Apparent
Bias-corrected
Ideal
B= 200 repetitions, boot
Mean absolute error=0.006 n=490

(E) Model 5

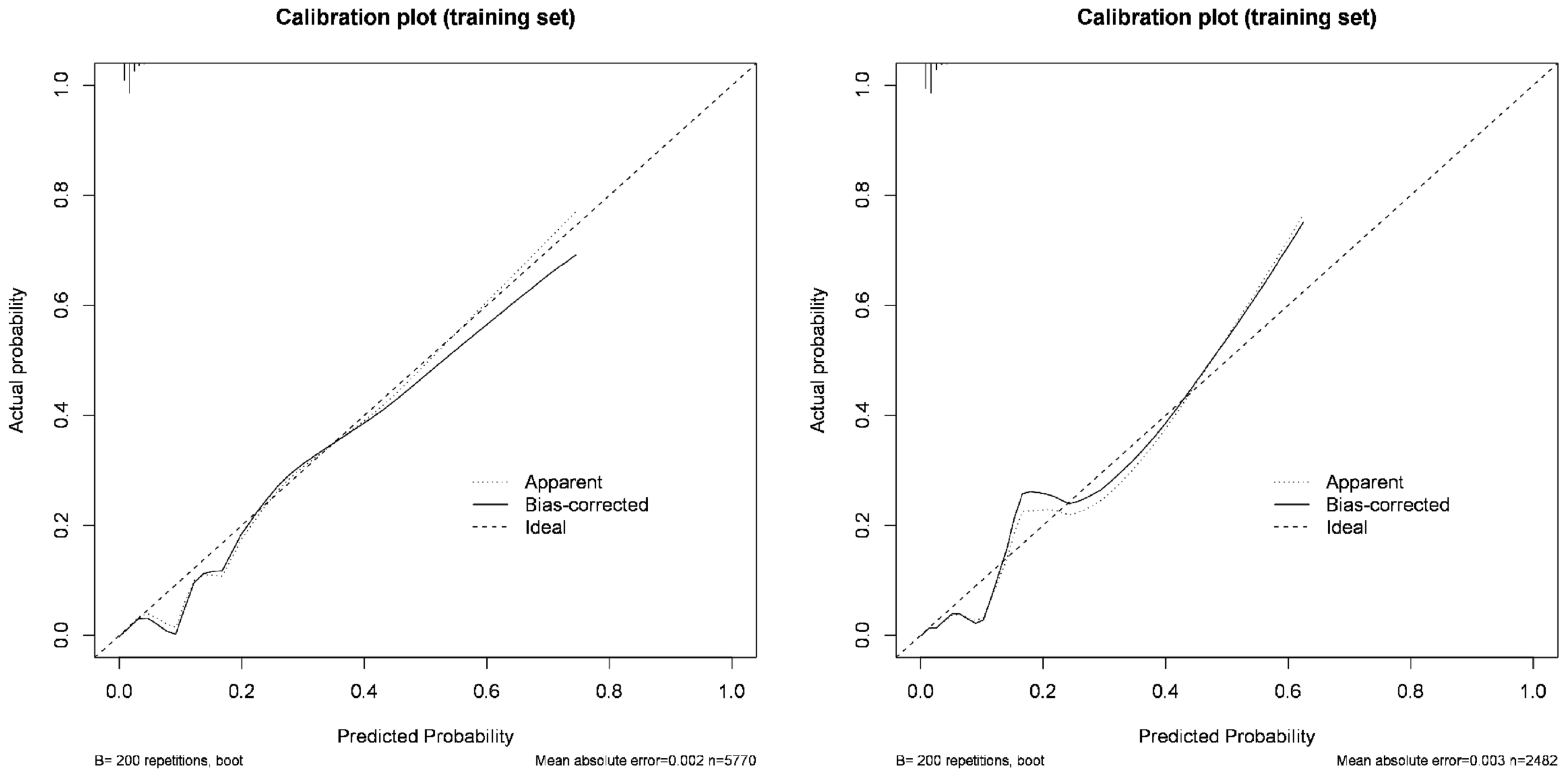
Calibration plot (training set)
Actual probability
Predicted Probability
0.0
0.2
0.4
0.6
0.8
1.0
Apparent
Bias-corrected
Ideal
B= 200 repetitions, boot
Mean absolute error=0.002 n=5770
Calibration plot (training set)
Actual probability
Predicted Probability
Apparent
Bias-corrected
Ideal
B= 200 repetitions, boot
Mean absolute error=0.003 n=2482

(F) Model 6

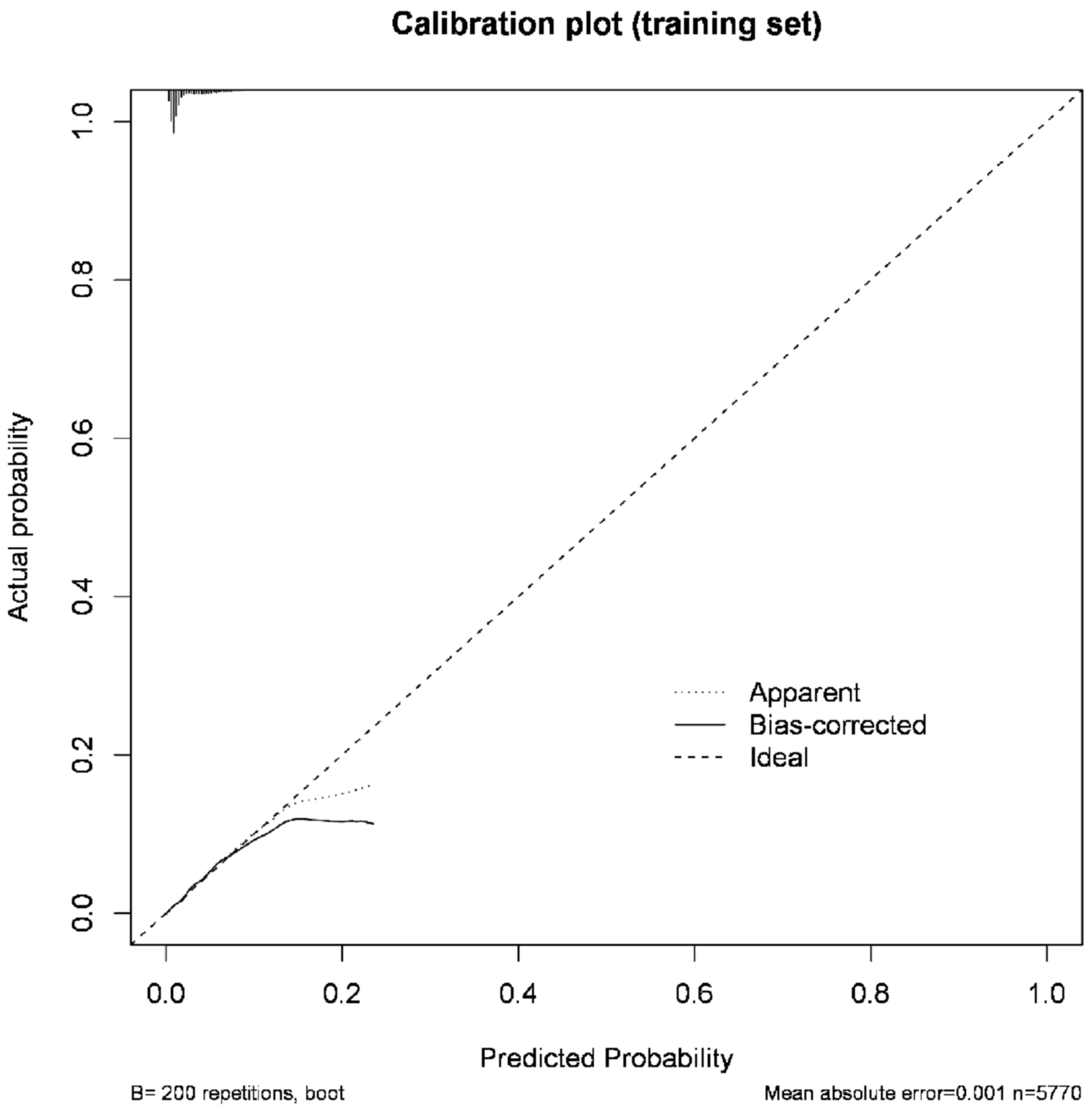
Calibration plot (training set)
Actual probability
Predicted Probability
Apparent
Bias-corrected
Ideal
B= 200 repetitions, boot
Mean absolute error=0.001 n=5770

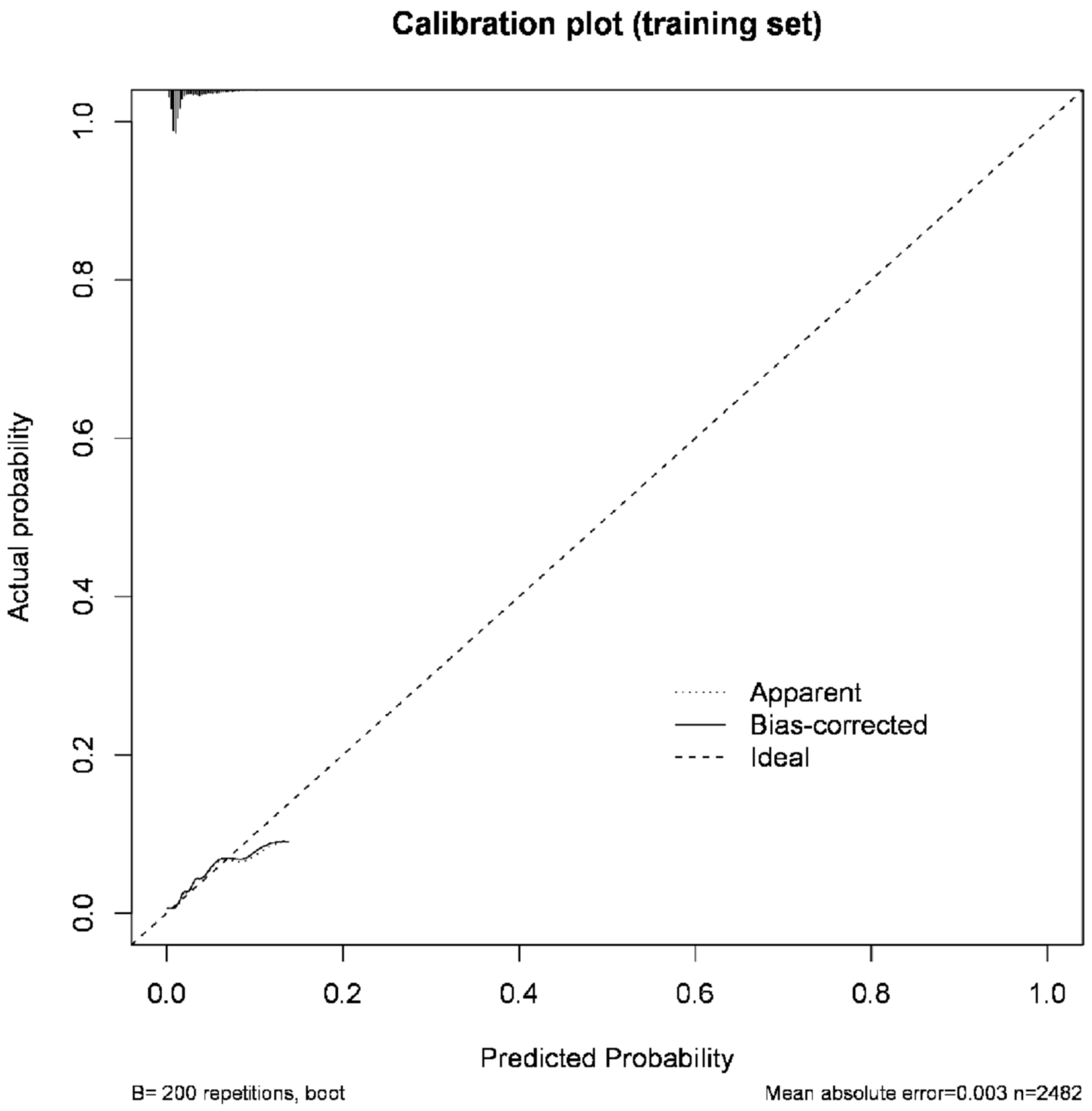
Calibration plot (training set)
Actual probability
Predicted Probability
Apparent
Bias-corrected
Ideal
B= 200 repetitions, boot
Mean absolute error=0.003 n=2482

(G) Model 7

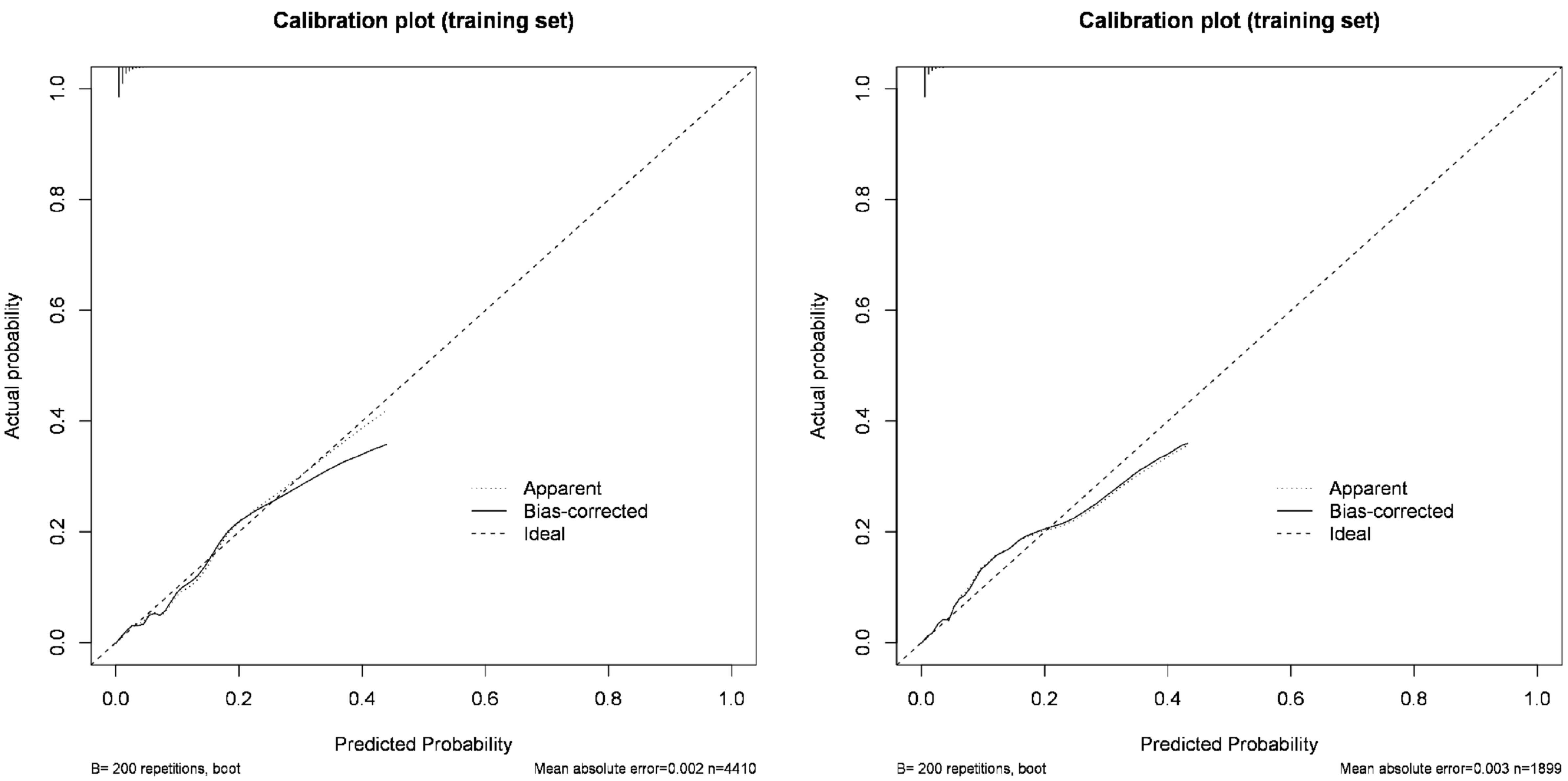

Calibration plot (training set)
Actual probability
Predicted Probability
0.0
0.2
0.4
0.6
0.8
1.0
Apparent
Bias-corrected
Ideal
B= 200 repetitions, boot
Mean absolute error=0.002 n=4410
Calibration plot (training set)
Actual probability
Predicted Probability
Apparent
Bias-corrected
Ideal
B= 200 repetitions, boot
Mean absolute error=0.003 n=1899

(H) Model 8

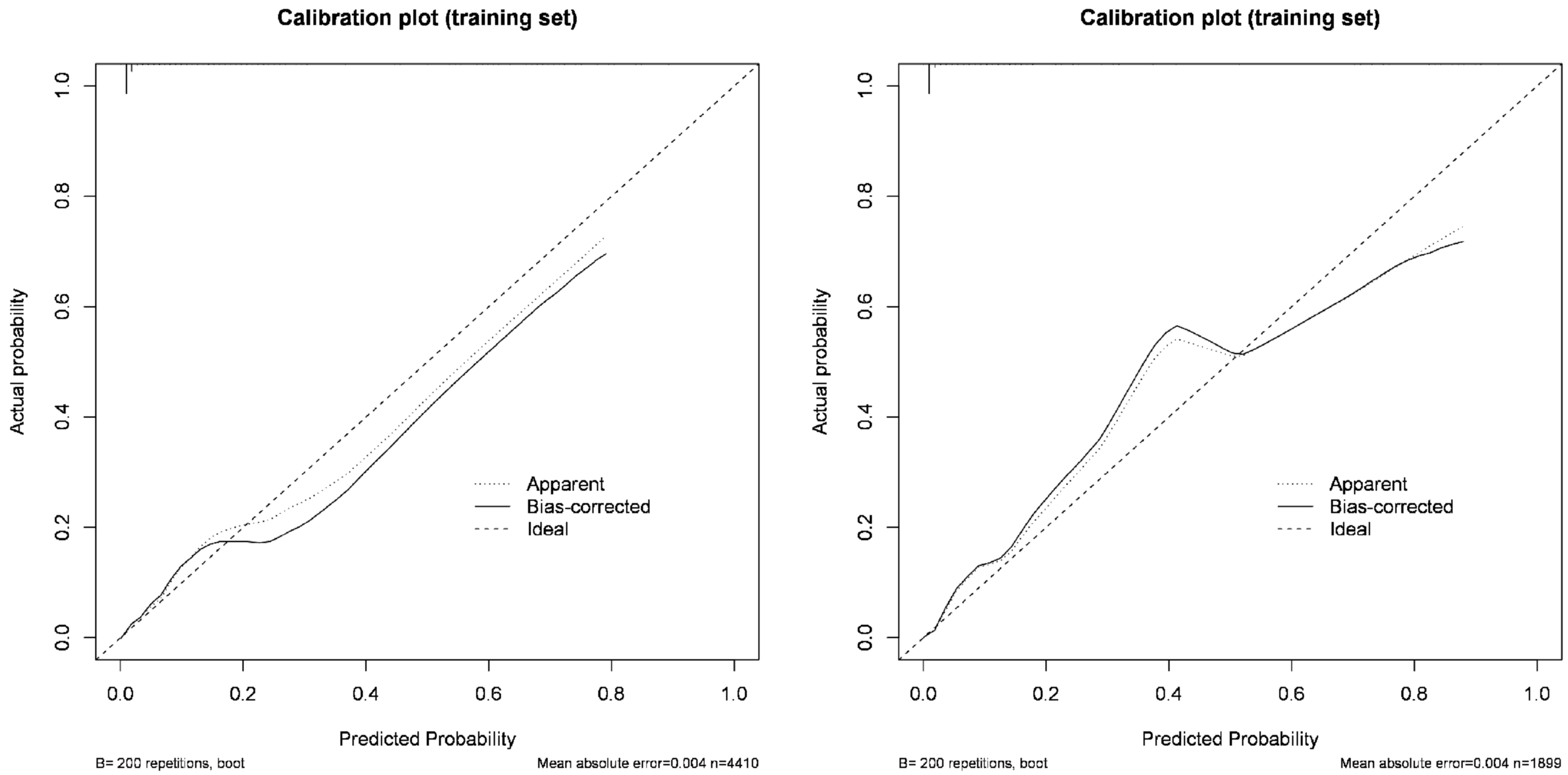
Calibration plot (training set)
Actual probability
Predicted Probability
Apparent
Bias-corrected
Ideal
0.0
0.2
0.4
0.6
0.8
1.0
B= 200 repetitions, boot
Mean absolute error=0.004 n=4410
Calibration plot (training set)
Actual probability
Predicted Probability
Apparent
Bias-corrected
Ideal
0.0
0.2
0.4
0.6
0.8
1.0
B= 200 repetitions, boot
Mean absolute error=0.004 n=1899

(I) Model 9

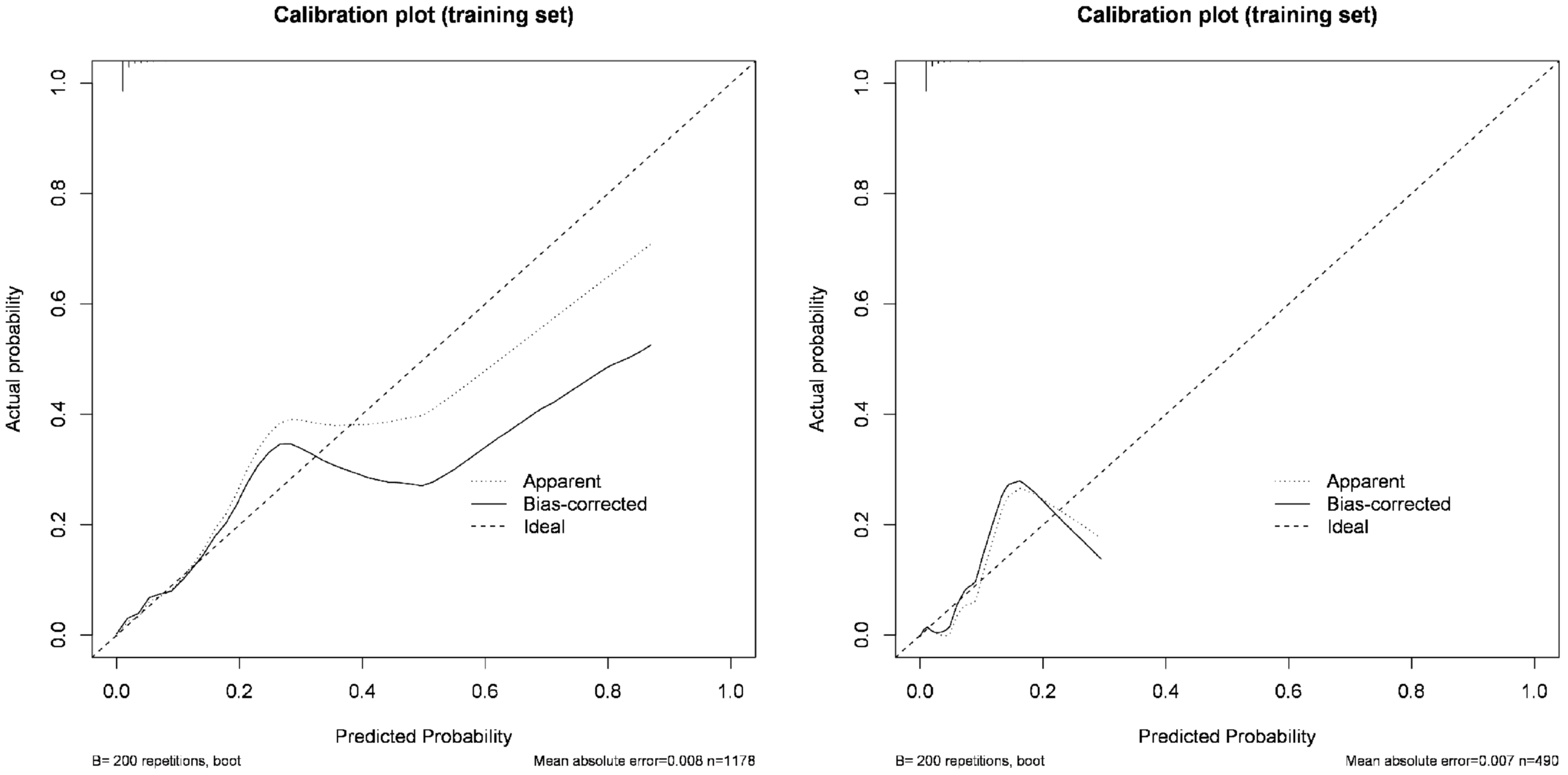
Calibration plot (training set)
Actual probability
0.0
0.2
0.4
0.6
0.8
1.0
Predicted Probability
Apparent
Bias-corrected
Ideal
B= 200 repetitions, boot
Mean absolute error=0.008 n=1178
Calibration plot (training set)
Actual probability
Predicted Probability
Apparent
Bias-corrected
Ideal
B= 200 repetitions, boot
Mean absolute error=0.007 n=490

(J) Model 10

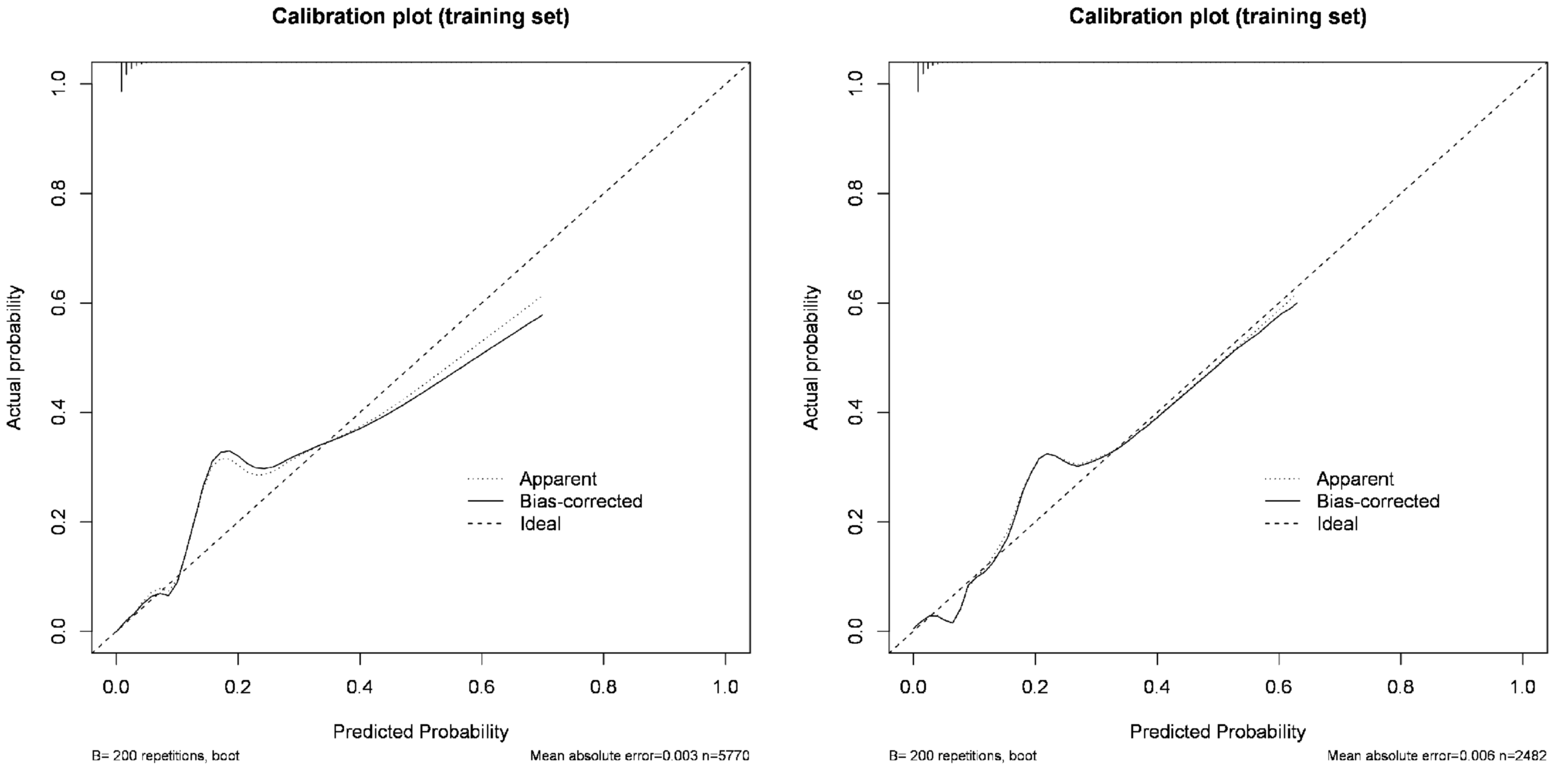
Calibration plot (training set)
Actual probability
Predicted Probability
0.0
0.2
0.4
0.6
0.8
1.0
Apparent
Bias-corrected
Ideal
B= 200 repetitions, boot
Mean absolute error=0.003 n=5770
Calibration plot (training set)
Actual probability
Predicted Probability
Apparent
Bias-corrected
Ideal
B= 200 repetitions, boot
Mean absolute error=0.006 n=2482

(K) Optimal diagnostic model

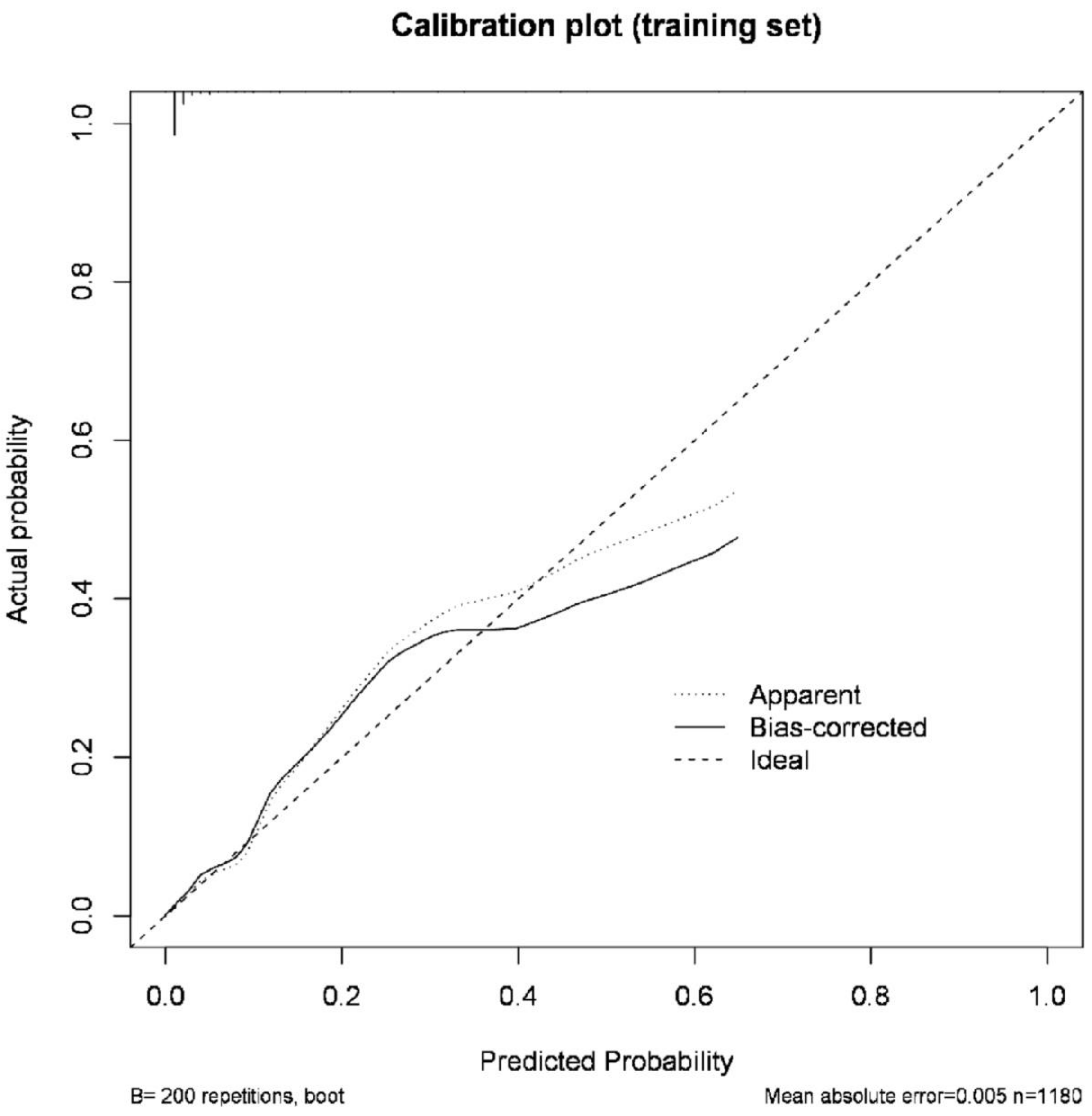

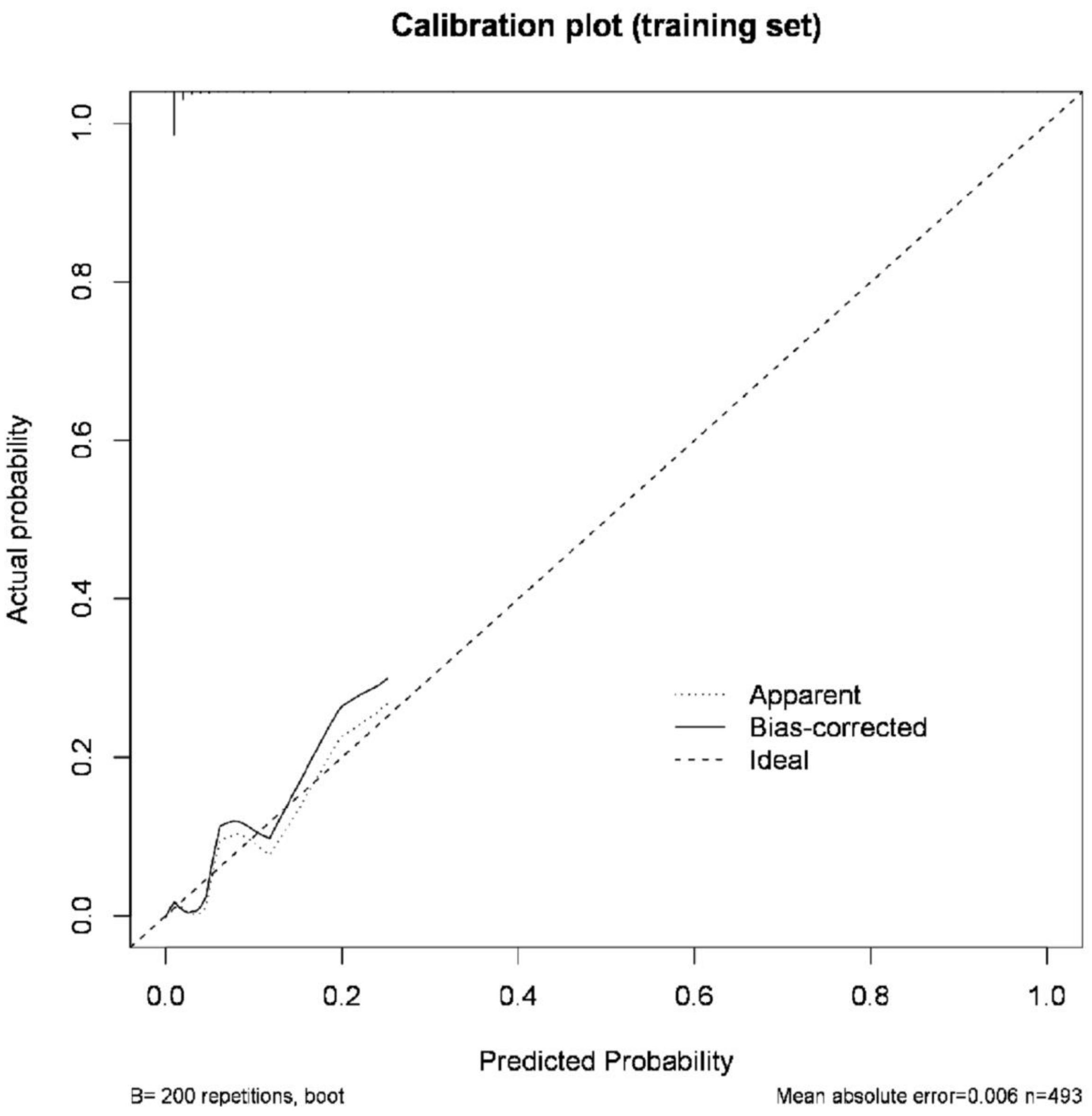

Supplement: Supplementary file 1 — Supplementary Figure S3. [file 41598_2023_47146_MOESM1_ESM.pdf]
