# Supplementary material for: Effect of multimodal diagnostic approach using deep learning-based automated detection algorithm for active pulmonary tuberculosis
Source: Sci Rep. 2023 Nov 13;13:19794. doi: 10.1038/s41598-023-47146-0 (PMC10643438; doi:10.1038/s41598-023-47146-0)
Supplement: Supplementary file 2 — Supplementary Table S1. [file 41598_2023_47146_MOESM2_ESM.docx]

Supplement Table S1. Unadjusted odds ratios for detecting pulmonary tuberculosis

| Variable | Univariable logistic regression | |
| --- | --- | --- |
|  | Odds ratio (95% CI) | P-value |
| Sex | 1.024 (0.702–1.494) | 0.903 |
| Age | 0.994 (0.983–1.005) | 0.306 |
| Mean arterial blood pressure | 1.005 (0.996–1.014) | 0.283 |
| Heart rate | 1.003 (0.995–1.011) | 0.480 |
| Respiratory rate | 0.898 (0.849–0.950) | <0.001 |
| Body temperature | 1.043 (0.865–1.258) | 0.656 |
| Saturation | 1.045 (1.000–1.091) | 0.052 |
| Hypertension | 0.956 (0.664–1.376) | 0.809 |
| Diabetes | 1.248 (0.851–1.829) | 0.256 |
| AIDS | 1.614 (0.218–11.931) | 0.639 |
| Old tuberculosis | 1.390 (0.862–2.240) | 0.176 |
| Alcohol history | 0.967 (0.653–1.433) | 0.868 |
| Smoking history | 0.782 (0.528–1.159) | 0.221 |
| Cough | 1.165 (0.810–1.677) | 0.410 |
| Sputum | 0.980 (0.677–1.417) | 0.913 |
| Fever | 1.198 (0.829–1.730) | 0.336 |
| Dyspnea | 0.720 (0.499–1.040) | 0.080 |
| Chest pain | 1.091 (0.689–1.728) | 0.711 |
| Hemoptysis | 1.160 (0.706–1.905) | 0.557 |
| Anorexia | 1.974 (1.021–3.818) | 0.043 |
| General weakness | 1.857 (1.128–3.057) | 0.015 |
| Sweating | 2.309 (0.308–17.306) | 0.416 |
| Weight loss | 5.431 (2.119–13.922) | <0.001 |
| Body mass index | 0.956 (0.882–1.036) | 0.270 |
| Albumin | 0.679 (0.505–0.914) | 0.011 |
| High density lipoprotein | 0.970 (0.928–1.014) | 0.175 |
| Low density lipoprotein | 0.996 (0.979–1.014) | 0.690 |
| Serum sodium | 0.958 (0.922–0.995) | 0.028 |
| Serum potassium | 0.876 (0.606–1.267) | 0.483 |
| Serum chloride | 0.975 (0.940–1.011) | 0.170 |
| Chest radiography | 11.714 (3.881–35.356) | <0.001 |
| Chest computed tomography | 21.254 (13.675–33.033) | <0.001 |
| Smear microscopy | 59.291 (38.286–91.819) | <0.001 |
| Polymerase chain reaction^a^ | 219.479 (43.331–1111.693) | <0.001 |
| TB screening score by DLAD | 5.922 (3.976–8.820) | <0.001 |

^a^ Polymerase chain reaction (Gene Xpert MTB/RIF)

CI, Confidence Interval; AIDS, Acquired Immune Deficiency Syndrome; TB, Tuberculosis; DLAD, Deep Learning-based Automated Detection algorithm
