# Supplementary material for: Effect of multimodal diagnostic approach using deep learning-based automated detection algorithm for active pulmonary tuberculosis
Source: Sci Rep. 2023 Nov 13;13:19794. doi: 10.1038/s41598-023-47146-0 (PMC10643438; doi:10.1038/s41598-023-47146-0)
Supplement: Supplementary file 3 — Supplementary Table S2. [file 41598_2023_47146_MOESM3_ESM.docx]

Supplement Table S2. Adjusted odds ratios in the 10 nested multicomponent diagnostic models

| Variable | Model 1 | | Model 2 | | Model 3 | | Model 4 | | Model 5 | |
| --- | --- | --- | --- | --- | --- | --- | --- | --- | --- | --- |
|  | Odds ratio  (95% CI) | P-value | Odds ratio  (95% CI) | P-value | Odds ratio  (95% CI) | P-value | Odds ratio  (95% CI) | P-value | Odds ratio  (95% CI) | P-value |
| Respiratory rate | 0.906  (0.853–0.961) | 0.001 | 0.916  (0.850–0.986) | 0.019 | 0.951  (0.881–1.025) | 0.188 | 0.933 (0.793–1.098) | 0.404 | 0.912 (0.856–0.973) | 0.005 |
| Saturation | 1.028  (0.980–1.078) | 0.261 | 1.014  (0.956–1.076) | 0.637 | 1.018 (0.956–1.085) | 0.573 | 1.349 (1.071–1.700) | 0.011 | 1.026 (0.975–1.080) | 0.323 |
| Dyspnea | 0.924  (0.612–1.397) | 0.709 | 0.975  (0.589–1.615) | 0.923 | 0.943  (0.545–1.631) | 0.835 | 0.902 (0.326–2.500) | 0.843 | 0.923 (0.586–1.455) | 0.731 |
| Anorexia | 1.253  (0.611–2.568) | 0.538 | 1.010  (0.376–2.714) | 0.984 | 0.829  (0.271–2.535) | 0.743 | 1.654 (0.289–9.447) | 0.572 | 1.008 (0.437–2.326) | 0.984 |
| General weakness | 1.474  (0.859–2.529) | 0.159 | 1.110  (0.556–2.218) | 0.767 | 1.542  (0.751–3.165) | 0.238 | 2.279 (0.564–9.203) | 0.248 | 1.673 (0.923–3.032) | 0.090 |
| Weight loss | 5.084  (1.948–13.267) | 0.001 | 4.669  (1.417–15.384) | 0.011 | 4.768  (1.333–17.055) | 0.016 | 6.074 (0.608–60.659) | 0.124 | 4.468 (1.402–14.235) | 0.011 |
| Albumin | 0.642  (0.473–0.870) | 0.004 | 0.608  (0.425–0.869) | 0.006 | 0.686  (0.464–1.015) | 0.059 | 0.985 (0.466–2.083) | 0.968 | 0.660 (0.474–0.919) | 0.014 |
| Serum sodium | 0.960  (0.923–0.998) | 0.038 | 0.953  (0.907–1.000) | 0.051 | 0.956  (0.906–1.008) | 0.097 | 0.983 (0.887–1.090) | 0.749 | 0.969 (0.927–1.012) | 0.152 |
| Smear microscopy |  |  |  |  | 20.721  (11.125–38.595) | <0.001 | 8.059 (1.779–36.504) | 0.007 | 54.782 (34.529–86.915) | <0.001 |
| Polymerase chain reaction^a^ |  |  |  |  |  |  | 95.089 (1.685–5367.256) | 0.027 |  |  |
| Chest radiography | 14.571  (4.591–46.248) | <0.001 | 2.074  (0.618–6.960) | 0.238 | 0.822  (0.187–3.616) | 0.795 | 0.192 (0.000–152.345) | 0.628 | 2.324 (0.490–11.027) | 0.288 |
| TB screening score by DLAD |  |  |  |  |  |  |  |  |  |  |
| Chest computed tomography |  |  | 20.096  (12.668–31.880) | <0.001 | 11.679  (6.992–19.510) | <0.001 | 9.691 (3.640–25.803) | <0.001 |  |  |
|  |  |  |  |  |  |  |  |  |  |  |
| Variable | Model 6 | | Model 7 | | Model 8 | | Model 9 | | Model 10 | |
|  | Odds ratio  (95% CI) | P-value | Odds ratio  (95% CI) | P-value | Odds ratio  (95% CI) | P-value | Odds ratio  (95% CI) | P-value | Odds ratio  (95% CI) | P-value |
| Respiratory rate | 0.907 (0.854–0.963) | 0.002 | 0.923 (0.857–0.994) | 0.033 | 0.955 (0.885–1.030) | 0.235 | 0.936 (0.794–1.105) | 0.436 | 0.919 (0.862–0.980) | 0.010 |
| Saturation | 1.028 (0.978–1.079) | 0.276 | 1.012 (0.953–1.074) | 0.697 | 1.016 (0.953–1.082) | 0.630 | 1.340 (1.072–1.674) | 0.010 | 1.026 (0.973–1.081) | 0.350 |
| Dyspnea | 0.856 (0.565–1.296) | 0.463 | 0.881 (0.530–1.465) | 0.625 | 0.885 (0.510–1.536) | 0.665 | 0.876 (0.309–2.485) | 0.804 | 0.887 (0.560–1.404) | 0.608 |
| Anorexia | 1.232 (0.598–2.540) | 0.571 | 0.985 (0.362–2.676) | 0.976 | 0.858 (0.287–2.566) | 0.784 | 1.404 (0.242–8.138) | 0.705 | 1.093 (0.484–2.467) | 0.831 |
| General weakness | 1.432 (0.829–2.473) | 0.198 | 1.071 (0.531–2.163) | 0.848 | 1.503 (0.726–3.109) | 0.272 | 2.224 (0.518–9.544) | 0.282 | 1.688 (0.932–3.058) | 0.084 |
| Weight loss | 3.565 (1.330–9.557) | 0.012 | 3.376 (1.005–11.342) | 0.049 | 3.975 (1.120–14.108) | 0.033 | 5.016 (0.563–44.712) | 0.148 | 3.598 (1.151–11.245) | 0.028 |
| Albumin | 0.644 (0.474–0.875) | 0.005 | 0.589 (0.410–0.846) | 0.004 | 0.660 (0.444–0.979) | 0.039 | 0.915 (0.425–1.968) | 0.820 | 0.644 (0.462–0.899) | 0.010 |
| Serum sodium | 0.977 (0.938–1.017) | 0.252 | 0.965 (0.918–1.015) | 0.171 | 0.965 (0.914–1.018) | 0.194 | 0.982 (0.885–1.090) | 0.731 | 0.983 (0.941–1.028) | 0.457 |
| Smear microscopy |  |  |  |  | 16.346 (8.809–30.331) | <0.001 | 6.656 (1.508–29.384) | 0.012 | 37.629 (23.455–60.367) | <0.001 |
| Polymerase chain reaction^a^ |  |  |  |  |  |  | 84.770 (3.435–2091.687) | 0.007 |  |  |
| Chest radiography |  |  |  |  |  |  |  |  |  |  |
| TB screening score by DLAD | 5.645 (3.776–8.440) | <0.001 | 3.376 (2.026–5.627) | <0.001 | 2.514 (1.459–4.330) | 0.001 | 3.637 (1.245–10.628) | 0.018 | 3.522 (2.274–5.455) | <0.001 |
| Chest computed tomography |  |  | 14.466 (9.000–23.251) | <0.001 | 9.035 (5.350–15.260) | <0.001 | 6.357 (2.281–17.717) | <0.001 |  |  |

^a^ Polymerase chain reaction (Gene Xpert MTB/RIF)

CI, Confidence Interval; TB, Tuberculosis; DLAD, Deep Learning-based Automated Detection algorithm
